# Irregular Sleep/Wake Patterns Are Associated With Reduced Quality of Life in Post-treatment Cancer Patients: A Study Across Three Cancer Cohorts

**DOI:** 10.3389/fnins.2021.700923

**Published:** 2021-09-22

**Authors:** Ritu Trivedi, Hong Man, Ayey Madut, Marius Mather, Elisabeth Elder, Haryana M. Dhillon, Alison Brand, Julie Howle, Graham Mann, Anna DeFazio, Terence Amis, Sean W. Cain, Andrew J. K. Phillips, Kristina Kairaitis

**Affiliations:** ^1^Ludwig Engel Centre for Respiratory Research, The Westmead Institute for Medical Research, Westmead, NSW, Australia; ^2^Sydney Informatics Hub, The University of Sydney, Camperdown, NSW, Australia; ^3^Westmead Clinical School, Faculty of Medicine and Health, The University of Sydney, Sydney, NSW, Australia; ^4^Westmead Breast Cancer Institute, Westmead Hospital, Westmead, NSW, Australia; ^5^Centre for Medical Psychology and Evidence-Based Decision-Making, School of Psychology, Faculty of Science, The University of Sydney, Camperdown, NSW, Australia; ^6^Psycho-Oncology Cooperative Research Group, School of Psychology, Faculty of Science, The University of Sydney, Camperdown, NSW, Australia; ^7^Department of Gynecological Oncology, Westmead Hospital, Westmead, NSW, Australia; ^8^Crown Princess Mary Cancer Centre, Westmead and Blacktown Hospitals, Sydney, NSW, Australia; ^9^Melanoma Institute Australia, The University of Sydney, Sydney, NSW, Australia; ^10^Centre for Cancer Research, The Westmead Institute for Medical Research, Westmead, NSW, Australia; ^11^Sydney West Translational Cancer Research Centre, Westmead, NSW, Australia; ^12^Department of Respiratory and Sleep Medicine, Westmead Hospital, Westmead, NSW, Australia; ^13^Turner Institute for Brain and Mental Health, School of Psychological Sciences, Monash University, Clayton, VIC, Australia

**Keywords:** sleep quality, sleep regularity, quality of life, cancer, cancer symptoms, endometrial cancer, breast cancer, melanoma

## Abstract

**Background:** Cancer patients often describe poor sleep quality and sleep disruption as contributors to poor quality of life (QoL). In a cross-sectional study of post-treatment breast, endometrial, and melanoma cancer patients, we used actigraphy to quantify sleep regularity using the sleep regularity index (SRI), and examined relationships with reported sleep symptoms and QoL.

**Methods:** Participants were recruited post-primary treatment (35 diagnosed with breast cancer, 24 endometrial cancer, and 29 melanoma) and wore an actigraphy device for up to 2 weeks and SRI was calculated. Self-report questionnaires for cancer-related QoL [European Organization for Research and Treatment of Cancer EORTC (QLQ-C30)] were completed. Data were compared using analysis of variance (ANOVA) or Chi-Square tests. Multivariate linear regression analysis was used to determine independent variable predictors for questionnaire-derived data.

**Results:** Age distribution was similar between cohorts. Endometrial and breast cancer cohorts were predominantly female, as expected, and body mass index (BMI) was higher in the endometrial cancer cohort, followed by breast and melanoma. There were no differences between tumor groups in: total sleep time, sleep onset latency, bedtime, and SRI (breast 80.9 ± 8.0, endometrial 80.3 ± 12.2, and melanoma 81.4 ± 7.0) (all *p* > 0.05). A higher SRI was associated with both better functional and symptom scores, including increased global QoL, better physical functioning, less sleepiness and fatigue, better sleep quality, and associated with less nausea/vomiting, dyspnea, and diarrhea (all *p* < 0.05).

**Conclusion:** In cancer patients post-treatment, greater sleep regularity is associated with increased global QoL, as well as better physical functioning and fewer cancer related symptoms. Improving sleep regularity may improve QoL for cancer patients.

## Introduction

Sleep disturbance is a common symptom associated with cancer and its treatment, with up to 95% of people with cancer (Fortner et al., 2002; Langford et al., 2012) reporting disturbed sleep. Sleep disturbance has a negative impact on quality of life (QoL) in this population (Sun and Lin, 2016). For some people with cancer, the negative impact of poor sleep persists post-treatment (Ancoli-Israel et al., 2014) and has been associated with poorer cancer outcomes and QoL. In women with a history of breast cancer, perceived poor sleep quality post-treatment is a predictor for a poorer survival outcome (Palesh et al., 2014). Long sleep duration and frequent sleep difficulties are associated with increased breast cancer mortality in women with a history of breast cancer (Palesh et al., 2014; Trudel-Fitzgerald et al., 2017). Understanding and addressing sleep problems in people with cancer may result in improvements in long-term well-being and survival.

Sleep is often assessed using subjective metrics derived from questionnaires designed to probe an individual’s perception of their at-home sleep experience over time. Actigraphy is a well-established, non-invasive, objective methodology for evaluating sleep across a variety of settings including cancer populations (Smith et al., 2018). Actigraphic metrics are highly correlated with gold-standard sleep assessment *via* polysomnography (PSG; Madsen, 2015). Actigraphy studies of cancer populations have usually quantified sleep in terms of sleep duration and sleep efficiency (Fernandes et al., 2006; Ancoli-Israel et al., 2014; Lévi et al., 2014; Palesh et al., 2014; Palesh, 2017). However, these metrics do not adequately capture other dimensions of sleep disturbance, including day-to-day variability in sleep patterns (Bei et al., 2016).

The sleep regularity index (SRI; Phillips et al., 2017) is a recently developed sleep metric that quantifies the consistency of an individual’s daily sleep/wake pattern by computing the percentage probability of an individual being in the same state (sleep vs. wake) at any two time-points 24 h apart, averaged across the study period. The metric effectively measures the degree of overlap in sleep/wake patterns between consecutive days, with values theoretically ranging from 0 (random) to 100 (completely regular). In practice, SRI values typically range from approximately 30 to 95, with a smaller number indicating less regular sleep (Phillips et al., 2017). Recent studies have reported that, compared with the standard actigraphy metrics of sleep duration and sleep efficiency, SRI has a stronger association with increased cardiometabolic risk (Lunsford-Avery et al., 2018), poorer mood (Pye et al., 2021), and poorer academic performance (Phillips et al., 2017).

Sleep/wake patterns in people with cancer are characterized by fragmented sleep, difficulty falling and staying asleep, waking up earlier than intended, unrestorative sleep (Roth, 2007), daytime napping, and low activity during the day (Davidson, 2002; Arndt, 2006; Kotronoulas, 2012; Lowery-Allison, 2018). These sleep patterns are likely to result in day-to-day variability in sleep/wake patterns, and contribute to a lower SRI score. Mood disorders (Mitchell et al., 2011), and cardiovascular death are common among cancer survivors due to shared risk factors (Sturgeon et al., 2019). Despite the recognition of increased mood disorders, cardiovascular risk, subjective poor sleep, and sleep fragmentation in cancer populations, SRI has not been quantified in any cancer population. In addition, the association with QoL in any population has not been examined. The aim of this study was to quantify the SRI, across three post-treatment cancer cohorts (breast, endometrial, and melanoma) using actigraphy and examine associations with subjective measurements of QoL and sleep. We hypothesized that irregular sleep/wake patterns in patients with a history of cancer, quantified using the SRI would be associated with a reduced QoL, and to subjective sleep.

## Materials and Methods

The study was approved by the Western Sydney Local Health District Ethics Committee (AU RED HREC/15/WMEAD/369). Written, informed consent was obtained from all participants.

### Participants

Participants were recruited from either breast (*n* = 35), endometrial (*n* = 24), or melanoma (*n* = 28) outpatient cancer clinics at Westmead Hospital between 2017 and 2019. Potential participants were approached in person by the investigators, and invited to participate in a study on sleep in cancer. Participants were included if they: (1) were at least 18 years of age; (2) had a confirmed diagnosis of breast cancer, endometrial cancer, or melanoma; and (3) had completed treatment (e.g., chemotherapy, radiotherapy, or surgery) either 2 months (endometrial cancer), 3 months (melanoma) or 12 months (breast cancer) previously. Timing of recruitment was chosen to mitigate against the acute impacts of a recent diagnosis or treatment regime. Participants were excluded if they: (1) had any serious or active medical or psychiatric comorbidities that would likely interfere with their assessment, or compliance with the protocol; (2) were unable/unlikely to comply with the study requirements for any reason; or (3) were pregnant. Twenty one of the breast cancer patients and 17 of the endometrial cancer patients had participated in a previous study (Madut et al., 2021).

### Data Collection and Study Procedures

We collected demographic data, menopausal status, cancer history, including histopathology and treatment, and current medications from participants medical records. We did not collect information on sleeping environment.

#### Questionnaires

Subjective sleep symptoms and sleep quality were assessed at recruitment using the following validated questionnaires: (1) Pittsburgh Sleep Quality Index (Carpenter, 1998; Backhaus, 2002; Beaudreau, 2012) (PSQI), (2) Epworth Sleepiness Scale (Johns, 1992) (ESS), and (3) Insomnia Severity Index (Savard et al., 2005) (ISI). QoL was assessed using the validated European Organization for Research and Treatment of Cancer Quality of Life Core (Fayers, 2002) (EORTC QLQ-C30) questionnaire. The EORTC QLQ-30 produces subscales of Global QoL, Functional scales (physical functioning, role functioning, emotional functioning, cognitive functioning, and social functioning) and Symptom scales (fatigue, nausea and vomiting, pain, dyspnoea, insomnia, appetite loss, constipation, diarrhea, and financial difficulties).

#### Actigraphy

Participants wore an *Actiwatch-2* (Philips Respironics, Bend, OR, United States) on their non-dominant wrist for up to 14 consecutive days, except when showering. Data were downloaded into proprietary software (Actiware, Phillips Respironics, Bend, OR, United States) for analysis. Automatically analyzed data for 30-s epochs were reviewed, quality checked, and manually adjusted. Rest periods were identified as the absence of light with continued activity, and sleep time identified as reduction in both light and activity. Time periods where the actiwatch was not worn by the participant with a complete absence of activity were excluded from analysis. Total sleep time, sleep onset latency, sleep effectiveness, wake after sleep onset (WASO), number of awakenings, get up, and bedtime were determined using the proprietary software algorithms.

### Sleep Regularity Index Calculation

The SRI was calculated using the established method (Phillips et al., 2017). The SRI was calculated as SRI = 200×Agreement/Cases−100, where Agreement is the number of valid (non-missing) epochs 24 h apart in the same sleep/wake state across the actigraphy recording, while Cases is the number of valid (non-missing) epochs 24 h apart. Patients were not included for SRI calculation if there were fewer than 5 days (120 h) of valid overlapping epochs of actigraphy (*n* = 6 patients). Five valid overlapping days is the minimum required data to obtain an accurate estimate of sleep regularity (Fischer et al., 2021).

### Statistical Analyses

Statistical analyses were conducted using R version 3.6.2. *P* < 0.05 was considered significant. Univariate comparisons were performed using ANOVA for continuous variables and Chi-Squared Tests for categorical variables. Multivariate analysis was performed using linear regression models to determine independent variable predictors for questionnaire-derived data (dependent variables). Each of the subscales of the EORTC QLQ-C30 were included. Regression models were run using complete cases only. Actigraphy measures were entered into these models as potential predictors with log_10_ transformation for skewed variables (reported below). The regression models controlled for the covariates of age, BMI, neck circumference, gender, and cancer type. Bed time was coded with midnight as zero, with times between midday and midnight coded as negative values (number of h away from midnight), and those between midnight and midday as positive. All numeric predictors were mean centered and scaled. To avoid issues with collinearity, time in bed, and get up time were not included as predictors in these models, as they were linearly dependent on other variables already included (sleep onset time and wake time, respectively). Given the inherent correlation between some of the actigraphy measures, Variance Inflation Factors were also checked to diagnose collinearity (Johnston et al., 2018). The breast cancer cohort was used as the reference group for all linear regression models.

## Results

### Participant Demographics

Participant demographics are shown in Table 1. Average age was not significantly different between cancer cohorts (*p* = 0.489). As expected, endometrial and breast cancer cohorts were almost exclusively female. The majority of women were post-menopausal, apart from one endometrial cancer and four melanoma patients. Nineteen breast cancer patients were taking anti- oestrogen therapy (tamoxifen or an aromatase inhibitor). Of the melanoma cohort, 69% were male. Endometrial cancer patients had a higher BMI relative to the other groups (*p* = 0.001).

**TABLE 1:** Patient demographics.

Group		Breast cancer	Endometrial cancer	Melanoma	Total	*p*
*n*		35	24	29	88	
Gender *n* (%)	F	34 (97.1)	24 (100.0)	9 (31.0)	67 (76.1)	< 0.001
	M	1 (2.9)	0 (0.0)	20 (69.0)	21 (23.9)	
Mean (STD)						
Age (years)		62.1 (9.5)	62.5 (9.1)	59.5 (11.8)	61.3 (10.2)	0.49
BMI (kg/m^2^)		28.4 (5.7)	34.3 (7.4)	30.8 (4.7)	30.8 (6.3)	0.001

### Cancer Grade/Stage and Treatment Modalities and Sleep Assessment

Cancer grade/stage data and classification of treatment modalities are provided in Supplementary Table 1. Most of the breast cancer patients had Grade 2/3 malignancies treated with a combination of surgery, radiation, chemotherapy, and endocrine treatments. The majority of endometrial (Grade 1/Stage 1a) and melanoma participants (Stage Ia + Ib) had less advanced malignancies and were treated with surgery alone (hysterectomy and bilateral salpingo-oophorectomy and excision, respectively). For the breast cancer patients, actigraphy was performed an average of 2 years after initial diagnosis, endometrial cancer patients were assessed around 7 months after diagnosis and melanoma was assessed around 14 months after initial diagnosis (Supplementary Table 1).

### Standard Actigraphy Metrics

Data for standard actigraphy metrics for each of the three cohorts are shown in Table 2. Breast cancer participants had, on average, 6 and 14 min greater WASO than the endometrial and melanoma groups, respectively (*p* = 0.018). There were no other significant cancer cohort differences for other standard actigraphy metrics (all *p* > 0.22; Table 2).

**TABLE 2:** Standard actigraphy metrics.

Variable	Breast cancer	Endometrial cancer	Melanoma	*p*
*n*	35	24	29	
Mean (SD)				
Sleep time (h)	7.5(1.0)	7.3(0.8)	7.3(1.1)	0.701
Onset latency (min)	29.7(21.8)	28.8(18.2)	34.1(25.2)	0.634
Sleep efficiency (%)	82.4(5.3)	82.6(4.5)	82.6(7.4)	0.980
Wake after sleep onset (min)	54.5(20.9)	48.1(15.3)	41.6(15.7)	0.018
Number of awakenings (*n*)	39.5(11.5)	35.4(10.4)	35.1(11.3)	0.218
Getup time (h)	07:16(00:13)	07:03(00:18)	07:07(00:16)	0.727
Bed time (h)	22:12(00:19)	22:19(00:31)	22:05(00:37)	0.918

### Sleep Regularity Index

**FIGURE 1:**
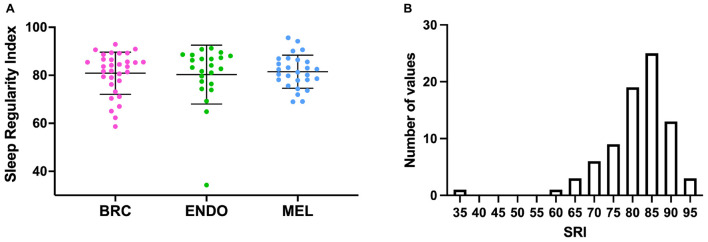
**(A)** Sleep Regularity Index (SRI) values for individual cancer patients (dots) by cancer cohort. BRC (pink), breast cancer, ENDO (green), endometrial cancer and MEL (blue), Melanoma. Bars indicate mean (SD). **(B)** Frequency histogram for SRI for all of the cancer cohorts.

Sleep regularity index could not be measured in six subjects (three breast cancer patients, one endometrial cancer patient, and two melanoma patients), as actigraphy data collection time was less than 5 overlapping days. For the 82 subjects in whom we measured SRI, actigraphy data for an average of 11.2 ± 2.0 days (range 5.9–13.2 days) was analyzed, and only one subject did not have a weekend day included. For those excluded from analysis, average actigraphy data collection was 3.46 ± 0.97 days (range 1.9–4.6 days). The SRI values ranged from 34.3 to 95.6, with left-skewed distributions, particularly in the endometrial and breast cancer cohorts (Figure 1). Group mean values for SRI were not significantly different (*p* = 0.09) between cancer cohorts (breast cancer: 80.9 ± 8.0; endometrial cancer: 80.3 ± 12.2; and melanoma cancer: 81.4 ± 7.0).

### Questionnaire Data

**FIGURE 2:**
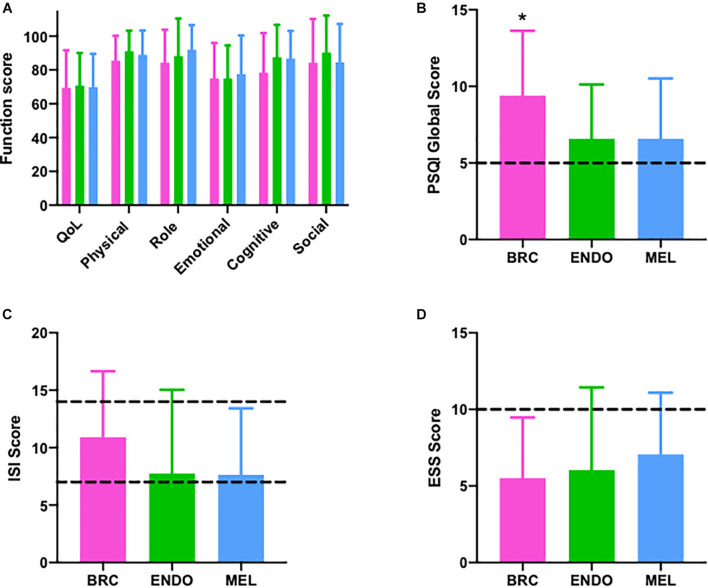
Questionnaire outcomes in breast (pink; *n* = 35), endometrial (green; *n* = 24), and melanoma (blue; *n* = 29) cancer patients. **(A)** European Organization for Research and Treatment of Cancer EORTC QLQ-C30) questionnaire with quality of life (QoL) and individual function scores (physical, role, emotional, cognitive, and social), **(B)** Pittsburgh Sleep Quality Index (PSQI) >5 indicates poor sleep quality **(C)** Insomnia Severity Index (ISI) (Dashed lines ISI > 8 sub-threshold insomnia, >14 clinical insomnia), **(D)** Epworth Sleepiness Scale (Dashed lines: ESS > 10 indicated daytimes sleepiness). BRC, breast cancer; ENDO, endometrial cancer; and MEL, melanoma cancer, *p < 0.009.

Data by cancer cohort for each questionnaire, including QoL, functional, and symptoms scores from the QLQ-C30 questionnaire, are shown in Figure 2 and Supplementary Table 2. There were no significant differences between the cancer cohorts for the majority of questionnaire results. However, the breast cancer cohort had a significantly higher PSQI score (*p* = 0.007) than endometrial or melanoma cancer cohorts (Figure 2B). The ISI score tended to be highest in the breast cancer cohort, although the difference was not significant (Figure 2C and Supplementary Table 2; *p* = 0.066). Similarly, sleep disturbance symptoms assessed *via* the QLQ-C30 sleep item were significantly higher for the breast cancer cohort (Supplementary Table 2; *p* = 0.012) compared with the endometrial or melanoma cohorts. No other questionnaire data were significantly different between cancer cohorts (all *p* > 0.20).

### Multivariate Linear Regression Models

Variables included were bedtime, sleep onset latency (log-transformed), WASO, number of awakenings (log-transformed), SRI, age, BMI, gender (male), endometrial cancer, and melanoma. Full statistical models are presented in Supplementary Tables 3–20, while results for significant predictor variables only are shown in Table 3 (all *p* < 0.05).

**TABLE 3:** Coefficients from linear regression models predicting questionnaire variables [European Organization for Research and Treatment of Cancer Quality of Life Core (EORTC QLQ-C30) quality of life (QoL) scores, functional scores, and symptoms scales; Pittsburgh Sleep Quality Index (PSQI); Insomnia Severity Index (ISI); and Epworth Sleepiness Scale (ESS)].

Variable	*b*	95% CI	*p*
**Quality of life (*n* = 79)**			
Sleep regularity index	10.28	4.86 to 15.69	< 0.001
Gender: male	31.73	10.49 to 52.97	0.0040
Melanoma	–14.63	−28.86 to −0.39	0.044
**Physical functioning (*n* = 80)**			
Sleep regularity index	6.55	2.88 to 10.22	< 0.001
Age (years)	–4.57	−7.78 to −1.36	0.0059
Endometrial cancer	12.30	4.81 to 19.78	0.0017
**Emotional functioning (*n* = 79)**			
Gender: male	31.26	7.14 to 55.39	0.012
**Fatigue (*n* = 80)**			
Sleep regularity index	–7.50	−12.76 to −2.24	0.0059
**Nausea/vomiting (*n* = 80)**			
Onset latency (log-transformed)	–3.63	−7.21 to −0.04	0.048
Sleep regularity index	–3.88	−7.64 to −0.11	0.044
**Dyspnoea (*n* = 80)**			
Sleep regularity index	–5.53	−10.92 to −0.13	0.045
**Insomnia (*n* = 80)**			
Gender: male	–37.01	−70.28 to −3.75	0.030
**Appetite loss (*n* = 80)**			
Sleep time (h)	4.55	0.16 to 8.94	0.042
**Constipation (*n* = 80)**			
Bed time	5.89	0.87 to 10.91	0.022
**Diarrhea (*n* = 79)**			
Sleep time (h)	2.97	0.25 to 5.69	0.033
Onset latency (log-transformed)	–6.18	−9.47 to −2.88	< 0.001
Sleep regularity index	–3.58	−6.96 to −0.21	0.037
Gender: male	–17.47	−30.7 to −4.25	0.010
**Financial difficulties (*n* = 79)**			
Onset latency (log-transformed)	–8.93	−15 to −2.86	0.0045
Endometrial cancer	–16.50	−29.21 to −3.8	0.012
**PSQI (*n* = 80)**			
Onset latency (log-transformed)	–1.14	−2.19 to −0.08	0.035
Sleep regularity index	–1.51	−2.62 to −0.4	0.0082
Endometrial cancer	–3.11	−5.37 to −0.85	0.0076
**ISI (*n* = 80)**			
Sleep regularity index	–2.77	−4.45 to −1.09	0.0016
Gender: male	–6.90	−13.49 to −0.31	0.041
Endometrial cancer	–4.17	−7.59 to −0.74	0.018
**ESS (*n* = 80)**			
Bedtime	–1.15	−2.08 to −0.22	0.016
Sleep regularity index	–2.86	−3.96 to −1.76	< 0.001

*Only significant predictors are shown; for complete results, refer to Supplementary Tables 3–20. Numeric predictors were centered and scaled. Coefficients reflect the increase in scores for a one standard deviation increase in the predictor for continuous variables, or difference from the reference level for categorical variables, holding other variables constant.*

#### QLQ-C30 Quality of Life, Function, and Symptom Scales

##### Quality of life score

**FIGURE 3:**
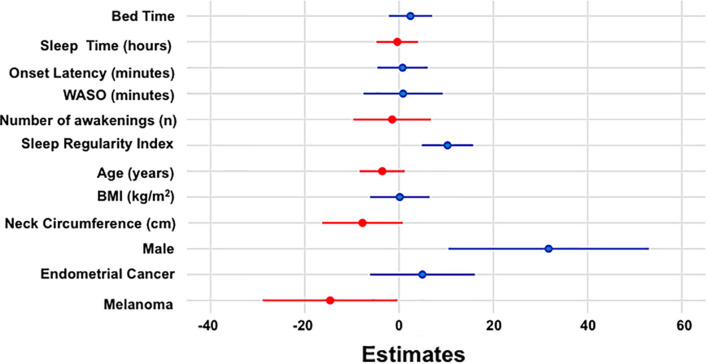
Estimated regression coefficients (dots) with 95% confidence intervals (bars) from linear regression model for QoL are shown with positive and negative coefficients represented in blue and red, respectively.

Sleep regularity index and male sex were associated with a higher QoL score, while being a member of the melanoma cohort was associated with a lower QoL score (see Table 3). No other independent variable was associated with QoL score (all *p* > 0.1; Supplementary Table 3). Figure 3 shows estimated regression coefficients for linear regression models for predictors of the QLQ-C30 QoL score.

##### Physical functioning score

Sleep regularity index and being a member of the endometrial cancer cohort were significantly positively associated with a higher Physical Functioning Score, while greater age was associated with a lower Physical Functioning Score (see Table 3). No other independent variable was associated with Physical Functioning Score (all *p* > 0.08; Supplementary Table 4).

##### Emotional functioning score

Male sex was associated with higher levels of Emotional Functioning (see Table 3). However, no other independent variable tested was associated with Emotional Functioning (all *p* > 0.07; Supplementary Table 6).

##### Symptom scores

Lower symptom scores for fatigue, nausea/vomiting, dyspnea, and diarrhea were associated with higher SRI values; while lower nausea/vomiting, diarrhea, and financial difficulty scores associated with increased sleep onset latency (log-transformed). Constipation was associated with a later bed time, while appetite loss, and diarrhea were associated with shorter total sleep time. Lower sleep and diarrhea symptoms scores were predicted in males, while lower scores for financial difficulties were in endometrial cancer (all *p* < 0.05, Table 3).

##### Role, cognitive, and social functioning scores

No independent variable tested was associated with the role, cognitive, and social functioning scores (all *p* > 0.07; Supplementary Tables 5, 7,8).

#### Pittsburgh Sleep Quality Index

Lower PSQI scores (better sleep quality) were predicted by a higher SRI score, a longer sleep onset latency, and in endometrial cancer cohorts. No other independent variable emerged as a significant predictor for the PSQI Score (all *p* > 0.09; Supplementary Table 18).

#### Insomnia Severity Index

Lower ISI scores (less insomnia symptoms) were predicted by a higher SRI value, male sex, and endometrial cancer (see Table 3). No other independent variable tested was a significant predictor for the ISI Score (all *p* > 0.1; Supplementary Table 19).

#### Epworth Sleepiness Scale

Lower ESS scores were predicted by later bedtimes and higher SRI values (see Table 3). No other independent variable tested emerged as a significant predictor for the ESS Score (all *p* > 0.2; Supplementary Table 19).

## Discussion

We found that greater sleep regularity is associated with higher QoL in melanoma, breast, and endometrial cancer patients. Sleep regularity was superior in predicting QoL relative to other actigraphic metrics of sleep, including sleep duration, sleep efficiency, and sleep timing. Greater sleep regularity was also a predictor of higher sleep quality, fewer insomnia symptoms, and less sleepiness. When the subscales of the cancer QoL questionnaires were examined, sleep regularity was also associated with better physical functioning, less fatigue, less dyspnoea and less nausea, and vomiting. These findings have important implications for assessing and improving sleep in cancer patients and may have implications for improving longer-term cancer survival.

In this group of cancer patients, the SRI was associated with many of the subjective cancer symptoms measured. Other predictors were also important, including: (1) male sex, which was associated with better QoL, emotional functioning, fewer diarrhea symptoms, and less insomnia severity; (2) shorter sleep onset latency, which was a predictor of less nausea and diarrhea symptoms, and fewer financial difficulties; (3) longer total sleep time, which was a predictor of more diarrhea symptoms and greater appetite loss; and (4) later sleep onset time, which was associated with less sleepiness. The relationship between less physical symptoms such as gastrointestinal symptoms, breathlessness, and better sleep regularity is a novel finding. These associations may be explained by the presence of circadian rhythms in all biological processes. Gastro-intestinal functions have clear circadian variation (Voigt et al., 2019), and similarly, breathlessness has demonstrated circadian rhythmicity (Tsai et al., 2007). The negative associations between nausea and vomiting, diarrhea and sleep onset latency may similarly be circadian rhythms, or alternatively sleep may be delayed due to gastrointestinal symptoms. Circadian rhythms and relationships to sleep regularity are discussed in more detail below. Relationship between sleep onset latency to financial difficulties would seem to be most likely a consequence of anxiety (Vahtera et al., 2007). No previous studies have examined the associations between regular sleep and cancer symptoms.

In addition to cancer symptoms and QoL, sleep regularity was also a better predictor of other sleep symptoms in these cancer patients than any other actigraphic metric of sleep. A higher SRI was associated with better sleep quality (as measured by the PSQI), less insomnia (as measured by the ISI), and less sleepiness (as measured by the ESS). Most participants slept for around 7 h with average sleep efficiency of 82%, similar to previous reports in breast (Berger and Higginbotham, 2000; Ancoli-Israel et al., 2006; Palesh et al., 2014) and endometrial cancer patients (Armbruster, 2018). However, the objective actigraphic measurement most closely predicting sleep symptoms was the SRI.

Sleep regularity has emerged recently as a powerful predictor of a range of health outcomes. Sleep regularity has not been measured in the general population, however, in an older group of healthy patients is reported to be around 86.4 (Pye et al., 2021). Our cancer cohorts have similar values for SRI to older patients with current depression (Pye et al., 2021). Recent studies have found lower SRI to be associated with depression (Pye et al., 2021), insomnia, post traumatic stress disorder (PTSD) (Mascaro et al., 2021), poorer mood (Sano et al., 2015), cardiometabolic dysfunction (Lunsford-Avery et al., 2018; Fritz et al., 2020), and poorer functional outcomes in autism (Cohen et al., 2017) and delayed sleep-wake phase disorder (Murray et al., 2019). The SRI has also been found to improve with treatment for alcohol dependence (Brooks et al., 2020). Our findings show that SRI is also a strong predictor for measures of QoL in cancer populations, including both functional and symptom scores. The broad utility of the SRI is likely due to its composite nature. Rather than being based on variability in any one dimension of sleep (e.g., variability in sleep onset time or total sleep time), the SRI compares the overall degree of overlap in sleep/wake patterns between consecutive days. Consequently, it is sensitive to many aspects of sleep disruption, including variation in sleep onset time, wake time, sleep duration, WASO, and napping. Given sleep disruption manifests in various forms across different health conditions, the SRI may have general clinical utility as a predictor of multiple outcomes.

The day-to-day variability of sleep/wake patterns is a consequence of factors including behavioral influences, social settings, circadian rhythms, and light sensitivity. Circadian rhythms are daily cycles that occur in virtually all biological processes, centrally co-ordinated and controlled by the brain’s master clock, the suprachiasmatic nucleus (SCN). Less regular sleep is associated with later onset of melatonin production (Phillips et al., 2017) and less stable timing of melatonin onset (Watson et al., 2020). Recent studies have shown significant variability in the population in light-related suppression of SCN outputs (Phillips et al., 2019), and home lighting exposures are in the range of light likely to suppress SCN outputs (Phillips et al., 2019; Cain et al., 2020). Our study has not examined which mechanisms are contributing in these cancer cohorts. This knowledge may lead to approaches to improve sleep regularity, which may in turn improve QoL, physical functioning, and cancer-related symptoms. Measurement of sleep regularity in cancer patients is relatively simple, uses standard clinical tools that are relatively available such as actigraphy. Addressing and improving sleep regularity may even result in better cancer outcomes in the longer term; at least in breast cancer patients sleep disruption and extreme sleep duration are associated with increased long-term mortality (Trudel-Fitzgerald et al., 2017). Whether improvements in sleep regularity will improve short or long term cancer outcomes is as yet unknown.

This is a relatively small study of a restricted number of cancer cohorts. The study is cross- sectional, and demonstrates associations rather than causation. An alternative interpretation may be that physical symptoms such as pain, breathlessness, and gastrointestinal disturbance can result in irregular sleep. In addition, the majority of participants were women, a group with more sleep-related symptoms, and all the male participants had melanoma. However, results for each of the symptom questionnaires, as well as actigraphy, were similar to those reported in larger, more diverse cancer populations. Actigraphy was analyzed without the assistance of a sleep diary. It has been demonstrated that there are small systematic differences in measured sleep duration and sleep onset latency when automatic analysis of actigraphy is compared with a sleep diary, resulting in slightly longer sleep duration with automatic analysis (van Hees et al., 2018). This may have impacted on the sleep regularity measurement, although as it is a systematic error this effect is unlikely to be large. Another important factor is that many of the patients may have had other sleep disorders, such as obstructive sleep apnea (OSA), that may have influenced sleep regularity and may be an important mediator of the effects of low SRI on outcomes in this population. In particular, we have recently demonstrated that nearly two-thirds of women with breast or endometrial cancer have moderate to severe OSA (Madut et al., 2021), which would be expected to associate with lower SRI due to frequent awakenings. Many of the patients in this study were also involved in this earlier study, and 41% all participants had a history of snoring. In addition, we did not collect information on sleeping environments such as bed partners, or employment status which may result in structured or unstructured days, and in turn have influenced sleep regularity. Despite these limitations, this small pilot study is suggestive of an important role for sleep regularity in QoL, physical functioning, and cancer-related symptoms in patients with a history of cancer.

In summary, in this novel investigation of sleep regularity in cancer cohorts, we found that sleep regularity is an important predictor for QoL. There is growing appreciation for considering other dimensions of sleep, including sleep regularity, in measuring sleep and circadian disruption, and predicting the downstream effects on health. Parallel to findings in other populations, sleep regularity was a stronger predictor of many key outcomes than other sleep-related metrics. These findings suggest we need to enrich traditional quota-based assessments of total sleep time and sleep efficiency with measures of sleep regularity for a fuller picture of health. Moreover, attention to measuring sleep regularity using widely available actigraphic methods including consumer devices, and management strategies aimed at improving sleep regularity (e.g., potentially *via* controlled lighting exposures) may provide a pathway to better sleep and QoL outcomes for cancer patients in the clinical setting.

## Data Availability Statement

The data analyzed for this study are available at: https://hdl.handle.net/2123/26161.

## Ethics Statement

The studies involving human participants were reviewed and approved by the Western Sydney Local Health District Ethics Committee. The patients/participants provided their written informed consent to participate in this study.

## Author Contributions

RT: data curation, formal analysis, and writing original draft. HM and AM: data curation and investigation. MM: formal analysis. EE, AB, JH, and AD: conceptualization, funding acquisition, resources, and writing review and editing. HD: conceptualization, funding acquisition, and writing review and editing. GM: conceptualization, funding acquisition, and resources. TA and KK: conceptualization, formal analysis, funding acquisition, investigation, methodology, project administration, supervision, and writing review and editing. AP and SC: conceptualization, formal analysis, methodology, and writing review and editing. All authors contributed to the article and approved the submitted version.

## Conflict of Interest

The authors declare that the research was conducted in the absence of any commercial or financial relationships that could be construed as a potential conflict of interest.

## Publisher’s Note

All claims expressed in this article are solely those of the authors and do not necessarily represent those of their affiliated organizations, or those of the publisher, the editors and the reviewers. Any product that may be evaluated in this article, or claim that may be made by its manufacturer, is not guaranteed or endorsed by the publisher.
