# Supplementary material for: Irregular Sleep/Wake Patterns Are Associated With Reduced Quality of Life in Post-treatment Cancer Patients: A Study Across Three Cancer Cohorts
Source: Front Neurosci. 2021 Sep 22;15:700923. doi: 10.3389/fnins.2021.700923 (PMC8494030; doi:10.3389/fnins.2021.700923)
Supplement: Supplementary file 1 [file Table_1.DOCX]

Supplementary Material

#### Supplementary Table S1. Cancer grade/stage and treatment modalities

| **Breast Cancer (n=35)** | | | |
| --- | --- | --- | --- |
|  | *Grade^a^* | | |
|  |  | Grade 1 | 7 (20%) |
|  |  | Grade 2 | 12 (34%) |
|  |  | Grade 3 | 14 (40%) |
|  |  | MISSING | 2 (6%) |
|  | *Surgery^b^ ± Adjuvant therapy* | | |
|  |  | Surgery + No adjuvant therapy | 3 |
|  |  | Surgery + Radiation | 5 |
|  |  | Surgery + Radiation + Chemotherapy | 4 |
|  |  | Surgery + Radiation + Endocrine therapy | 4 |
|  |  | Surgery + Radiation + Chemotherapy + Endocrine therapy | 17 |
|  |  | MISSING | 2 |
|  | *Time interval from diagnosis to sleep assessment* | | |
|  |  | Median (interquartile range): days | 691 (490-782) |
|  |  | Range: days | 366-1668 |
| **Endometrial Cancer (n=24)** | | | |
|  | *Grade^c^* | | |
|  |  | Grade 1 | 21 (88 %) |
|  |  | Grade 2 | 2 (8 %) |
|  |  | Grade 3 | 1 (4 %) |
|  |  | MISSING | 0 |
|  | *Stage^d^* |  |  |
|  |  | Stage Ia | 21 |
|  |  | Stage Ib | 3 |
|  |  | MISSING | 0 |
|  | *Surgery ± Adjuvant therapy* | | |
|  |  | Hysterectomy | 21 |
|  |  | Hysterectomy + Radiation therapy | 3 |
|  |  | MISSING | 0 |
|  | *Time interval from diagnosis to sleep assessment* | | |
|  |  | Median (interquartlle range): days | 222 (114-427) |
|  |  | Range : days | 59-886 |
| **Melanoma Cancer (n=29)** | | | |
|  | *Stage^e^* | | |
|  |  | Stage Ia + Ib | 15 (52%) |
|  |  | Stage IIa + IIb | 6 (21%) |
|  |  | Stage IIIIa + IIIb + IIIc | 3 (10%) |
|  |  | Stage IV | 1 (3%) |
|  |  | MISSING | 4 (14%) |
|  | *Surgery* | | |
|  |  | Wide local excision surgery | 26 |
|  |  | MISSING | 3 |
|  | *Time interval from diagnosis to sleep assessment* | | |
|  |  | Median (interquartlle range): days | 419 (299-930) |
|  |  | Range : days | 107-1924 |

^a^ breast cancer grade (criteria?)

^b^ breast cancer surgery

^c^ endometrial cancer grade, FIGO (International Federation of Gynecology and Obstetrics) system

^d^ endometrial cancer FIGO stage

^e^ melanoma stage AJCM

#### Supplementary Table S2. Questionnaire outcomes

| **Group** | **Missing N** | **Breast Cancer** | **Endometrial Cancer** | **Melanoma** | **Total** | **p** |
| --- | --- | --- | --- | --- | --- | --- |
| Total N |  | 35 | 24 | 29 | 88 |  |
| **Mean (SD)** |  |  |  |  |  |  |
| ***EORTC-QLQ C30*** |  |  |  |  |  |  |
| QoL | 1 | 69.4 (22.3) | 70.8 (19.2) | 69.8 (19.8) | 69.9 (20.4) | 0.965 |
| Cognitive functioning | 1 | 78.4 (23.4) | 87.5 (19.2) | 86.8 (16.3) | 83.7 (20.3) | 0.151 |
| Emotional functioning | 1 | 75.0 (21.0) | 75.0 (19.5) | 77.6 (22.8) | 75.9 (21.0) | 0.867 |
| Social functioning | 1 | 84.3 (25.9) | 90.3 (21.9) | 84.5 (22.7) | 86.0 (23.7) | 0.590 |
| Role functioning | 0 | 84.3 (19.4) | 88.2 (22.2) | 92.0 (14.5) | 87.9 (18.9) | 0.272 |
| Physical functioning | 0 | 85.5 (14.7) | 91.1 (12.2) | 89.0 (14.4) | 88.2 (14.0) | 0.305 |
| Fatigue | 0 | 26.3 (20.2) | 25.4 (21.6) | 21.4 (16.8) | 24.5 (19.4) | 0.586 |
| Nausea/Vomitting | 0 | 5.7 (14.5) | 7.0 (15.5) | 1.7 (5.2) | 4.7 (12.6) | 0.276 |
| Pain | 0 | 23.8 (28.7) | 16.0 (25.3) | 20.7 (24.7) | 20.6 (26.4) | 0.539 |
| Dyspnoea | 0 | 13.3 (20.1) | 12.5 (21.6) | 9.2 (17.6) | 11.7 (19.6) | 0.690 |
| Insomnia | 0 | 43.8 (33.1) | 27.8 (25.4) | 21.8 (28.6) | 32.2 (30.9) | 0.012 |
| Appetite loss | 0 | 10.5 (22.5) | 5.6 (21.2) | 3.4 (10.3) | 6.8 (19.0) | 0.318 |
| Constipation | 0 | 12.4 (19.9) | 12.5 (23.7) | 5.7 (15.6) | 10.2 (19.8) | 0.335 |
| Diarrhea | 1 | 4.9 (12.0) | 5.5 (12.7) | 4.6 (14.7) | 5.0 (13.0) | 0.965 |
| ***Global PSQI*** | 0 | 9.4 (4.2) | 6.6 (3.5) | 6.6 (3.9) | 7.7 (4.1) | 0.007 |
| ***ISI*** | 1 | 10.9 (5.7) | 7.8 (7.3) | 7.6 (5.8) | 9.0 (6.3) | 0.066 |
| ***ESS*** | 0 | 5.5 (4.0) | 6.0 (5.4) | 7.1 (4.0) | 6.2 (4.4) | 0.373 |

#### Supplementary Table S3. Coefficients from a linear regression model predicting QLQ-C30 QoL scores. Complete case n= 79. Numeric predictors were centered and scaled. Coefficients reflect the increase in scores for a 1 standard deviation increase in the predictor for continuous variables, or difference from the reference level for categorical variables.

| **Variable** | **b** | **95% CI** | **=p** |
| --- | --- | --- | --- |
| (Intercept) | 66.20 | 58.38 - 74.03 | < 0.001 |
| Bed time | 2.41 | -2.16 - 6.97 | 0.30 |
| Sleep time (hours) | -0.37 | -4.74 - 3.99 | 0.86 |
| Onset latency (log-transformed) | 0.73 | -4.56 - 6.03 | 0.78 |
| Wake After Sleep Onset | 0.84 | -7.55 - 9.24 | 0.84 |
| Number of awakenings (log-transformed) | -1.48 | -9.64 - 6.69 | 0.72 |
| Sleep Regularity Index | 10.28 | 4.86 - 15.69 | < 0.001 |
| Age (years) | -3.57 | -8.3 - 1.17 | 0.14 |
| BMI | 0.14 | -6.12 - 6.41 | 0.96 |
| Gender: Male | 31.73 | 10.49 - 52.97 | 0.0040 |
| Endometrial cancer | 4.95 | -6.13 - 16.03 | 0.38 |
| Melanoma | -14.63 | -28.86 - -0.39 | 0.044 |

**Supplementary Table S4.** Coefficients from a linear regression model predicting **QLQ-C30 physical functioning score.** Complete case n = 80. Numeric predictors were centered and scaled. Coefficients reflect the increase in scores for a 1 standard deviation increase in the predictor for continuous variables, or difference from the reference level for categorical variables.

| **Variable** | **b** | **95% CI** | **p** |
| --- | --- | --- | --- |
| (Intercept) | 81.47 | 76.21 – 86.73 | < 0.001 |
| Bed time | 0.72 | -2.37 – 3.82 | 0.64 |
| Sleep time (hours) | -1.07 | -4.03 – 1.89 | 0.47 |
| Onset latency (log-transformed) | 1.44 | -2.06 – 4.94 | 0.42 |
| Wake After Sleep Onset | 1.06 | -4.4 – 6.52 | 0.70 |
| Number of awakenings (log-transformed) | 2.42 | -2.83 – 7.67 | 0.36 |
| Sleep Regularity Index | 6.55 | 2.88 – 10.22 | < 0.001 |
| Age (years) | -4.57 | -7.78 - -1.36 | 0.0059 |
| BMI | -3.07 | -7.29 – 1.15 | 0.15 |
| Gender: Male | 12.17 | -2.23 – 26.57 | 0.096 |
| Endometrial cancer | 12.30 | 4.81 – 19.78 | 0.0017 |
| Melanoma | -0.08 | -9.72 – 9.55 | 0.99 |

#### Supplementary Table S5. Coefficients from a linear regression model predicting QLQ-C30 role functioning score. Complete case n = 80. Numeric predictors were centered and scaled. Coefficients reflect the increase in scores for a 1 standard deviation increase in the predictor for continuous variables, or difference from the reference level for categorical variables.

| **Variable** | **b** | **95% CI** | **p** |
| --- | --- | --- | --- |
| (Intercept) | 83.23 | 75.11 - 91.36 | < 0.001 |
| Bed time | -3.93 | -8.72 - 0.85 | 0.11 |
| Sleep time (hours) | -2.00 | -6.57 - 2.58 | 0.39 |
| Onset latency (log-transformed) | 0.85 | -4.56 - 6.26 | 0.76 |
| Wake After Sleep Onset | -1.88 | -10.31 - 6.56 | 0.66 |
| Number of awakenings (log-transformed) | 0.23 | -7.88 - 8.34 | 0.95 |
| Sleep Regularity Index | 3.75 | -1.92 - 9.43 | 0.19 |
| Age (years) | -2.32 | -7.29 - 2.64 | 0.35 |
| BMI | 0.84 | -5.68 - 7.37 | 0.80 |
| Gender: Male | 14.24 | -8.02 - 36.5 | 0.21 |
| Endometrial cancer | 4.82 | -6.75 - 16.39 | 0.41 |
| Melanoma | 0.65 | -14.24 - 15.54 | 0.93 |

#### Supplementary Table S6. Coefficients from a linear regression model predicting QLQ-C30 emotional functioning score. Complete case n = 79. Numeric predictors were centered and scaled. Coefficients reflect the increase in scores for a 1 standard deviation increase in the predictor for continuous variables, or difference from the reference level for categorical variables.

| **Variable** | **b** | **95% CI** | **p** |
| --- | --- | --- | --- |
| (Intercept) | 67.93 | 59.05 - 76.82 | < 0.001 |
| Bed time | -0.51 | -5.7 - 4.67 | 0.84 |
| Sleep time (hours) | -0.31 | -5.27 - 4.64 | 0.90 |
| Onset latency (log-transformed) | -5.20 | -11.21 - 0.81 | 0.089 |
| Wake After Sleep Onset | -0.63 | -10.17 - 8.9 | 0.89 |
| Number of awakenings (log-transformed) | 3.05 | -6.23 - 12.32 | 0.51 |
| Sleep Regularity Index | 5.52 | -0.63 - 11.67 | 0.078 |
| Age (years) | -0.68 | -6.06 - 4.69 | 0.80 |
| BMI | 1.00 | -6.11 - 8.12 | 0.78 |
| Gender: Male | 31.26 | 7.14 - 55.39 | 0.012 |
| Endometrial cancer | 7.44 | -5.15 - 20.03 | 0.24 |
| Melanoma | -7.75 | -23.93 - 8.42 | 0.34 |

**Supplementary Table S7.** Coefficients from a linear regression model **predicting QLQ-C30 cognitive functioning score**. Complete case n = 79. Numeric predictors were centered and scaled. Coefficients reflect the increase in scores for a 1 standard deviation increase in the predictor for continuous variables, or difference from the reference level for categorical variables.

| **Variable** | **b** | **95% CI** | **p** |
| --- | --- | --- | --- |
| (Intercept) | 76.29 | 67.85 - 84.73 | < 0.001 |
| Bed time | -3.99 | -8.91 - 0.93 | 0.11 |
| Sleep time (hours) | -1.23 | -5.94 - 3.48 | 0.60 |
| Onset latency (log-transformed) | -2.73 | -8.44 - 2.98 | 0.34 |
| Wake After Sleep Onset | -6.78 | -15.84 - 2.27 | 0.14 |
| Number of awakenings (log-transformed) | 3.54 | -5.27 - 12.35 | 0.43 |
| Sleep Regularity Index | 2.49 | -3.35 - 8.33 | 0.40 |
| Age (years) | -1.02 | -6.13 - 4.09 | 0.69 |
| BMI | 1.58 | -5.18 - 8.34 | 0.64 |
| Gender: Male | 20.57 | -2.35 - 43.49 | 0.078 |
| Endometrial cancer | 10.58 | -1.38 - 22.54 | 0.082 |
| Melanoma | -1.06 | -16.42 - 14.3 | 0.89 |

**Supplementary Table S8.** Coefficients from a linear regression model predicting **QLQ-C30 social functioning score**. Complete case n = 79. Numeric predictors were centered and scaled. Coefficients reflect the increase in scores for a 1 standard deviation increase in the predictor for continuous variables, or difference from the reference level for categorical variables.

| **Variable** | **b** | **95% CI** | **p** |
| --- | --- | --- | --- |
| (Intercept) | 85.02 | 74.88 - 95.17 | < 0.001 |
| Bed time | -4.91 | -10.83 - 1.01 | 0.10 |
| Sleep time (hours) | -1.44 | -7.1 - 4.22 | 0.61 |
| Onset latency (log-transformed) | 0.10 | -6.77 - 6.97 | 0.98 |
| Wake After Sleep Onset | -2.20 | -13.09 - 8.69 | 0.69 |
| Number of awakenings (log-transformed) | -2.50 | -13.09 - 8.09 | 0.64 |
| Sleep Regularity Index | 4.90 | -2.13 - 11.92 | 0.17 |
| Age (years) | -0.87 | -7.01 - 5.27 | 0.78 |
| BMI | 1.57 | -6.55 - 9.7 | 0.70 |
| Gender: Male | 16.07 | -11.48 - 43.63 | 0.25 |
| Endometrial cancer | 4.31 | -10.06 - 18.69 | 0.55 |
| Melanoma | -13.39 | -31.86 - 5.08 | 0.15 |

**Supplementary Table S9.** Coefficients from a linear regression model predicting **QLQ-C30** **Fatigue.** Complete case n = 80. Numeric predictors were centered and scaled. Coefficients reflect the increase in scores for a 1 standard deviation increase in the predictor for continuous variables, or difference from the reference level for categorical variables.

| **Variable** | **b** | **95% CI** | **p** |
| --- | --- | --- | --- |
| (Intercept) | 31.82 | 24.28 - 39.35 | < 0.001 |
| Bed time | 4.01 | -0.42 - 8.45 | 0.075 |
| Sleep time (hours) | 1.53 | -2.71 - 5.77 | 0.47 |
| Onset latency (log-transformed) | 2.02 | -3 - 7.04 | 0.42 |
| Wake After Sleep Onset | -0.50 | -8.32 - 7.32 | 0.90 |
| Number of awakenings (log-transformed) | -1.96 | -9.49 - 5.56 | 0.60 |
| Sleep Regularity Index | -7.50 | -12.76 - -2.24 | 0.0059 |
| Age (years) | 0.08 | -4.52 - 4.68 | 0.97 |
| BMI | 2.76 | -3.29 - 8.81 | 0.36 |
| Gender: Male | -16.56 | -37.2 - 4.08 | 0.11 |
| Endometrial cancer | -9.06 | -19.79 - 1.67 | 0.097 |
| Melanoma | -3.19 | -17 - 10.62 | 0.65 |

**Supplementary Table S10.** Coefficients from a linear regression model predicting **QLQ-C30** **Nausea/Vomiting.** Complete case n = 80. Numeric predictors were centered and scaled. Coefficients reflect the increase in scores for a 1 standard deviation increase in the predictor for continuous variables, or difference from the reference level for categorical variables.

| **Variable** | **b** | **95% CI** | **p** |
| --- | --- | --- | --- |
| (Intercept) | 4.66 | -0.73 - 10.05 | 0.089 |
| Bed time | -1.28 | -4.45 - 1.9 | 0.42 |
| Sleep time (hours) | -2.50 | -5.53 - 0.54 | 0.10 |
| Onset latency (log-transformed) | -3.63 | -7.21 - -0.04 | 0.048 |
| Wake After Sleep Onset | 1.77 | -3.82 - 7.37 | 0.53 |
| Number of awakenings (log-transformed) | 1.57 | -3.81 - 6.95 | 0.56 |
| Sleep Regularity Index | -3.88 | -7.64 - -0.11 | 0.044 |
| Age (years) | 0.64 | -2.65 - 3.93 | 0.70 |
| BMI | -0.12 | -4.45 - 4.2 | 0.95 |
| Gender: Male | -2.14 | -16.9 - 12.63 | 0.77 |
| Endometrial cancer | 2.30 | -5.38 - 9.97 | 0.55 |
| Melanoma | 0.55 | -9.33 - 10.43 | 0.91 |

**Supplementary Table S11.** Coefficients from a linear regression model predicting QLQ-C30 **Pain**. Complete case n = 80. Numeric predictors were centered and scaled. Coefficients reflect the increase in scores for a 1 standard deviation increase in the predictor for continuous variables, or difference from the reference level for categorical variables.

| **Variable** | **b** | **95% CI** | **p** |
| --- | --- | --- | --- |
| (Intercept) | 22.92 | 12.08 - 33.76 | < 0.001 |
| Bed time | 0.55 | -5.83 - 6.94 | 0.86 |
| Sleep time (hours) | 1.47 | -4.64 - 7.57 | 0.63 |
| Onset latency (log-transformed) | -6.78 | -14 - 0.44 | 0.065 |
| Wake After Sleep Onset | -2.74 | -13.99 - 8.52 | 0.63 |
| Number of awakenings (log-transformed) | 7.77 | -3.05 - 18.6 | 0.16 |
| Sleep Regularity Index | -7.15 | -14.72 - 0.43 | 0.064 |
| Age (years) | 2.07 | -4.55 - 8.69 | 0.54 |
| BMI | 1.85 | -6.86 - 10.55 | 0.67 |
| Gender: Male | -21.97 | -51.67 - 7.73 | 0.14 |
| Endometrial cancer | -6.98 | -22.42 - 8.46 | 0.37 |
| Melanoma | 15.28 | -4.6 - 35.15 | 0.13 |

**Supplementary Table S12.** Coefficients from a linear regression model predicting **QLQ-C30** **Dyspnoea**. Complete case n = 80. Numeric predictors were centered and scaled. Coefficients reflect the increase in scores for a 1 standard deviation increase in the predictor for continuous variables, or difference from the reference level for categorical variables.

| **Variable** | **b** | **95% CI** | **p** |
| --- | --- | --- | --- |
| (Intercept) | 13.77 | 6.04 - 21.49 | < 0.001 |
| Bed time | 0.78 | -3.76 - 5.33 | 0.73 |
| Sleep time (hours) | 1.17 | -3.17 - 5.52 | 0.59 |
| Onset latency (log-transformed) | -1.41 | -6.55 - 3.73 | 0.59 |
| Wake After Sleep Onset | 6.78 | -1.24 - 14.79 | 0.096 |
| Number of awakenings (log-transformed) | -5.51 | -13.22 - 2.2 | 0.16 |
| Sleep Regularity Index | -5.53 | -10.92 - -0.13 | 0.045 |
| Age (years) | 2.15 | -2.56 - 6.87 | 0.37 |
| BMI | 4.98 | -1.22 - 11.18 | 0.11 |
| Gender: Male | -1.90 | -23.06 - 19.25 | 0.86 |
| Endometrial cancer | -4.33 | -15.33 - 6.66 | 0.43 |
| Melanoma | -2.57 | -16.72 - 11.59 | 0.72 |

**Supplementary Table S13.** Coefficients from a linear regression model predicting **QLQ-C30** **Sleep.** Complete case n = 80. Numeric predictors were centered and scaled. Coefficients reflect the increase in scores for a 1 standard deviation increase in the predictor for continuous variables, or difference from the reference level for categorical variables.

| **Variable** | **b** | **95% CI** | **p** |
| --- | --- | --- | --- |
| (Intercept) | 44.16 | 32.02 - 56.31 | < 0.001 |
| Bed time | 0.14 | -7.01 - 7.29 | 0.97 |
| Sleep time (hours) | 4.61 | -2.22 - 11.45 | 0.18 |
| Onset latency (log-transformed) | -7.11 | -15.2 - 0.97 | 0.084 |
| Wake After Sleep Onset | 10.58 | -2.03 - 23.18 | 0.099 |
| Number of awakenings (log-transformed) | -0.57 | -12.69 - 11.56 | 0.93 |
| Sleep Regularity Index | -5.95 | -14.43 - 2.53 | 0.17 |
| Age (years) | -0.55 | -7.96 - 6.87 | 0.88 |
| BMI | -4.12 | -13.87 - 5.63 | 0.40 |
| Gender: Male | -37.01 | -70.28 - -3.75 | 0.030 |
| Endometrial cancer | -14.13 | -31.42 - 3.17 | 0.11 |
| Melanoma | 1.85 | -20.4 - 24.11 | 0.87 |

**Supplementary Table S14.** Coefficients from a linear regression model predicting **QLQ-C30** **Appetite loss**. Complete case n = 80. Numeric predictors were centered and scaled. Coefficients reflect the increase in scores for a 1 standard deviation increase in the predictor for continuous variables, or difference from the reference level for categorical variables.

| **Variable** | **b** | **95% CI** | **p** |
| --- | --- | --- | --- |
| (Intercept) | 10.36 | 2.56 - 18.15 | 0.010 |
| Bed time | 3.26 | -1.32 - 7.85 | 0.16 |
| Sleep time (hours) | 4.55 | 0.16 - 8.94 | 0.042 |
| Onset latency (log-transformed) | 1.30 | -3.89 - 6.49 | 0.62 |
| Wake After Sleep Onset | -0.26 | -8.36 - 7.83 | 0.95 |
| Number of awakenings (log-transformed) | 6.15 | -1.63 - 13.93 | 0.12 |
| Sleep Regularity Index | -3.13 | -8.58 - 2.31 | 0.26 |
| Age (years) | 2.65 | -2.1 - 7.41 | 0.27 |
| BMI | -1.51 | -7.77 - 4.74 | 0.63 |
| Gender: Male | -15.44 | -36.79 - 5.91 | 0.15 |
| Endometrial cancer | -3.21 | -14.31 - 7.89 | 0.57 |
| Melanoma | 2.12 | -12.17 - 16.41 | 0.77 |

**Supplementary Table S15.** Coefficients from a linear regression model predicting **QLQ-C30** **Constipation**. Complete case n = 80. Numeric predictors were centered and scaled. Coefficients reflect the increase in scores for a 1 standard deviation increase in the predictor for continuous variables, or difference from the reference level for categorical variables.

| **Variable** | **b** | **95% CI** | **p** |
| --- | --- | --- | --- |
| (Intercept) | 15.52 | 6.99 - 24.05 | < 0.001 |
| Bed time | 5.89 | 0.87 - 10.91 | 0.022 |
| Sleep time (hours) | 1.54 | -3.26 - 6.35 | 0.52 |
| Onset latency (log-transformed) | 2.88 | -2.79 - 8.56 | 0.31 |
| Wake After Sleep Onset | -1.78 | -10.64 - 7.07 | 0.69 |
| Number of awakenings (log-transformed) | 0.56 | -7.96 - 9.07 | 0.90 |
| Sleep Regularity Index | -1.13 | -7.08 - 4.83 | 0.71 |
| Age (years) | -0.20 | -5.41 - 5 | 0.94 |
| BMI | -0.42 | -7.27 - 6.42 | 0.90 |
| Gender: Male | -8.84 | -32.21 - 14.52 | 0.45 |
| Endometrial cancer | -2.65 | -14.8 - 9.49 | 0.66 |
| Melanoma | -8.69 | -24.32 - 6.94 | 0.27 |

**Supplementary Table S16.** Coefficients from a linear regression model predicting **QLQ-C30** **Diarrhea.** Complete case n = 79. Numeric predictors were centered and scaled. Coefficients reflect the increase in scores for a 1 standard deviation increase in the predictor for continuous variables, or difference from the reference level for categorical variables.

| **Variable** | **b** | **95% CI** | **p** |
| --- | --- | --- | --- |
| (Intercept) | 5.06 | 0.19 - 9.93 | 0.042 |
| Bed time | -0.95 | -3.79 - 1.89 | 0.51 |
| Sleep time (hours) | 2.97 | 0.25 - 5.69 | 0.033 |
| Onset latency (log-transformed) | -6.18 | -9.47 - -2.88 | < 0.001 |
| Wake After Sleep Onset | 0.65 | -4.58 - 5.87 | 0.81 |
| Number of awakenings (log-transformed) | 2.66 | -2.42 - 7.75 | 0.30 |
| Sleep Regularity Index | -3.58 | -6.96 - -0.21 | 0.037 |
| Age (years) | 2.03 | -0.91 - 4.98 | 0.17 |
| BMI | -1.46 | -5.36 - 2.43 | 0.46 |
| Gender: Male | -17.47 | -30.7 - -4.25 | 0.010 |
| Endometrial cancer | 1.55 | -5.35 - 8.45 | 0.66 |
| Melanoma | 8.30 | -0.56 - 17.16 | 0.066 |

**Supplementary Table S17.** Coefficients from a linear regression model predicting **QLQ-C30** **Financial difficulties.** Complete case n = 79. Numeric predictors were centered and scaled. Coefficients reflect the increase in scores for a 1 standard deviation increase in the predictor for continuous variables, or difference from the reference level for categorical variables.

| **Variable** | **b** | **95% CI** | **p** |
| --- | --- | --- | --- |
| (Intercept) | 18.35 | 9.38 - 27.32 | < 0.001 |
| Bed time | -1.24 | -6.47 - 3.99 | 0.64 |
| Sleep time (hours) | -3.25 | -8.25 - 1.75 | 0.20 |
| Onset latency (log-transformed) | -8.93 | -15 - -2.86 | 0.0045 |
| Wake After Sleep Onset | -3.98 | -13.6 - 5.64 | 0.41 |
| Number of awakenings (log-transformed) | 3.59 | -5.77 - 12.95 | 0.45 |
| Sleep Regularity Index | -5.23 | -11.44 - 0.97 | 0.097 |
| Age (years) | -2.81 | -8.23 - 2.62 | 0.31 |
| BMI | 5.01 | -2.17 - 12.19 | 0.17 |
| Gender: Male | 2.32 | -22.03 - 26.67 | 0.85 |
| Endometrial cancer | -16.50 | -29.21 - -3.8 | 0.012 |
| Melanoma | -12.99 | -29.31 - 3.33 | 0.12 |

**Supplementary Table S18.** Coefficients from a linear regression model predicting **Pittsburgh Sleep Quality Index**. Complete case n = 80. Numeric predictors were centered and scaled. Coefficients reflect the increase in scores for a 1 standard deviation increase in the predictor for continuous variables, or difference from the reference level for categorical variables.

| **Variable** | **b** | **95% CI** | **p** |
| --- | --- | --- | --- |
| (Intercept) | 9.68 | 8.09 - 11.26 | < 0.001 |
| Bed time | 0.02 | -0.92 - 0.95 | 0.97 |
| Sleep time (hours) | 0.66 | -0.24 - 1.55 | 0.15 |
| Onset latency (log-transformed) | -1.14 | -2.19 - -0.08 | 0.035 |
| Wake After Sleep Onset | 0.10 | -1.54 - 1.75 | 0.90 |
| Number of awakenings (log-transformed) | 0.48 | -1.1 - 2.06 | 0.54 |
| Sleep Regularity Index | -1.51 | -2.62 - -0.4 | 0.0082 |
| Age (years) | -0.06 | -1.03 - 0.9 | 0.89 |
| BMI | 0.09 | -1.19 - 1.36 | 0.89 |
| Gender: Male | -3.62 | -7.96 - 0.73 | 0.10 |
| Endometrial cancer | -3.11 | -5.37 - -0.85 | 0.0076 |
| Melanoma | -0.45 | -3.36 - 2.45 | 0.76 |

**Supplementary Table S19.** Coefficients from a linear regression model predicting **Insomnia Severity Index**. Complete case n = 80. Numeric predictors were centered and scaled. Coefficients reflect the increase in scores for a 1 standard deviation increase in the predictor for continuous variables, or difference from the reference level for categorical variables.

| **Variable** | **b** | **95% CI** | **p** |
| --- | --- | --- | --- |
| (Intercept) | 12.09 | 9.68 - 14.5 | < 0.001 |
| Bed time | -0.61 | -2.03 - 0.8 | 0.39 |
| Sleep time (hours) | 0.72 | -0.64 - 2.07 | 0.29 |
| Onset latency (log-transformed) | -1.18 | -2.78 - 0.43 | 0.15 |
| Wake After Sleep Onset | -0.23 | -2.73 - 2.27 | 0.85 |
| Number of awakenings (log-transformed) | 0.21 | -2.19 - 2.62 | 0.86 |
| Sleep Regularity Index | -2.77 | -4.45 - -1.09 | 0.0016 |
| Age (years) | -1.17 | -2.64 - 0.3 | 0.12 |
| BMI | -0.42 | -2.35 - 1.52 | 0.67 |
| Gender: Male | -6.90 | -13.49 - -0.31 | 0.041 |
| Endometrial cancer | -4.17 | -7.59 - -0.74 | 0.018 |
| Melanoma | -0.72 | -5.14 - 3.69 | 0.74 |

**Supplementary Table S20.** Coefficients from a linear regression model predicting **Epworth Sleepiness Scale score**. Complete case n = 80. Numeric predictors were centered and scaled. Coefficients reflect the increase in scores for a 1 standard deviation increase in the predictor for continuous variables, or difference from the reference level for categorical variables.

| **Variable** | **b** | **95% CI** | **p** |
| --- | --- | --- | --- |
| (Intercept) | 6.08 | 4.5 - 7.66 | < 0.001 |
| Bed time | -1.15 | -2.08 - -0.22 | 0.016 |
| Sleep time (hours) | 0.33 | -0.55 - 1.22 | 0.46 |
| Onset latency (log-transformed) | 0.00 | -1.05 - 1.05 | 1.0 |
| Wake After Sleep Onset | 0.34 | -1.3 - 1.98 | 0.68 |
| Number of awakenings (log-transformed) | -0.88 | -2.45 - 0.7 | 0.27 |
| Sleep Regularity Index | -2.86 | -3.96 - -1.76 | < 0.001 |
| Age (years) | 0.09 | -0.87 - 1.06 | 0.85 |
| BMI | 0.33 | -0.94 - 1.6 | 0.60 |
| Gender: Male | -0.59 | -4.91 - 3.74 | 0.79 |
| Endometrial cancer | -0.46 | -2.7 - 1.79 | 0.69 |
| Melanoma | 1.28 | -1.61 - 4.17 | 0.38 |
